# What Can Integrated Analysis of Morphological and Genetic Data Still Reveal about the *Anastrepha fraterculus* (Diptera: Tephritidae) Cryptic Species Complex?

**DOI:** 10.3390/insects10110408

**Published:** 2019-11-15

**Authors:** Leandro F. Prezotto, André L.P. Perondini, Vicente Hernández-Ortiz, Daniel Frías, Denise Selivon

**Affiliations:** 1 Departamento de Genética e Biologia Evolutiva, Instituto de Biociências, Universidade de São Paulo, São Paulo 05508-090, Brazil; leandroprezotto@yahoo.com.br (L.F.P.);; 2 Red de Interacciones Multitróficas, Instituto de Ecología A.C., Xalapa, Veracruz 91073, Mexico; vicente.hernandez@inecol.mx; 3 Instituto de Entomología, Universidad Metropolitana de Ciencias de la Educación, Santiago 3311, Chile; daniel.frias@umce.cl

**Keywords:** fruit flies, ITS1 sequences, ribosomal spacer, wing morphometry, phylogeny

## Abstract

The South American fruit fly *Anastrepha fraterculus* (Wiedemann) is a complex of cryptic species, the so-called “*Anastrepha fraterculus* complex”, for which eight morphotypes are currently recognized. A previous analysis of ITS1 in samples of the *Anastrepha fraterculus* complex, while revealing high distinctiveness among samples from different localities of South America, Central America, and Mexico, no direct association was made between sequence type and morphotype. In the present report, a correlated analysis of morphometry and ITS1 data involved individuals belonging to the same population samples. Although showing a low level of intra-populational nucleotide variability, the ITS1 analysis indicated numerous inter-population sequence type variants. Morphotypes identified by morphometric analysis based on female wing shape were highly concordant with ITS1 genetic data. The correlation of genetic divergence and morphological differences among the tested samples gives strong evidence of a robust dataset, thereby indicating the existence of various taxonomic species within the *A. fraterculus* complex. However, the data revealed genetic and morphological variations in some regions, suggesting that further analysis is still required for some geographic regions.

## 1. Introduction

The genus *Anastrepha* Schiner is endemic to the Neotropics and has been found to occur from the southern United States to northern Argentina and the Caribbean Islands [1]. The genus comprises about 250 valid species with 21 species groups that differ in terms of morphology [2,3,4]. Among the 34 species of the “*fraterculus* group”, some are considered economically damaging, such as *Anastrepha fraterculus* (Wiedemann). In the 1940s, variations in wing pattern along its wide geographic distribution, similar to that of the genus, lead to the assumption that cryptic species might be involved [5,6,7]. Since then, samples from the nominal *Anastrepha fraterculus* populations have been analyzed for other biological parameters and significant data on the existence of different species have been accumulated over time.

The first non-morphological evidence indicating that different species could be involved in the *Anastrepha fraterculus* complex came from a karyotypic analyses of specimens collected in Brazil and Mexico, revealing marked differences in sex chromosomes [8,9]. Further karyotypic studies [10] and an isozymic analysis [11] also identified differences among specimens collected in north-eastern and south-eastern Brazil. This was further corroborated by a still wider isozymic analysis with additional samples from Venezuela, Peru, Costa Rica, and Mexico [12]. Later, mitochondrial DNA analysis revealed a high degree of differentiation between Venezuelan and south-eastern Brazilian populations [13,14]. Phylogenetic inferences based on molecular markers, 16S [15], CO1 mitochondrial DNA [16], and the nuclear-gene period [17], pointed out additional differences in *A. fraterculus s.l*., thus giving support to the hypothesis that *Anastrepha fraterculus* actually includes a complex of cryptic species [11]. However, in these studies, different samples and/or distinct biological parameters were analyzed, making the recognition of distinct taxa difficult. For example, the karyotype [8,9] and isozyme [11,15] data were obtained from distinct population samples.

This difficulty was overcome after an integrated analysis of linear morphometry, genetics, and tests of reproductive isolation allowed the description of three biological entities within the *Anastrepha fraterculus* (AF) complex in Brazil: *Anastrepha* sp.1 aff. *fraterculus*, *A.* sp.2. aff. *fraterculus*, and *A.* sp.3 aff. *fraterculus* (abbreviated as *A.* sp.1, *A.* sp.2 and *A.* sp.3) [18,19,20,21,22,23,24,25,26].

Apart from the description of the three species from Brazil and the evidences of genetic differences in samples of *A. fraterculus* along its geographic distribution, a linear morphometric approach applied to samples from Mexico, Colombia, Brazil, and Argentina provided evidence of distinct morphotypes occurring in the different regions [27]. The analysis was extended to other population samples [28,29], resulting in the characterization of eight morphotypes: “Mexican” (comprising samples from México, Guatemala, and Panama), “Venezuelan” (lowlands of Venezuela), “Andean” (highlands of Venezuela and Colombia), “Peruvian” (lowlands of Peru and Ecuador-*A*. sp.4), “Ecuadorian” (highlands of Peru and Ecuador), and three Brazilian morphotypes—“Brazilian-1” (including samples from Argentina), “Brazilian-2”, and “Brazilian-3”, corresponding to *A*. sp.1, *A*. sp.2 and *A*. sp.3, which have already been characterized [19,20]. However, except for the Brazilian samples, correlated analyses of other biological parameters were not included in the morphological studies. A huge international effort was done in order to characterize cryptic species complexes of tephritid fruit flies employing integrative analysis similar to that applied to Brazilian morphotypes. The result of this coordinate project was the recognition of eight morphotypes as distinct biological entities, and the review of these data may be accessed in [30].

The recognition of distinct morphotypes in the Neotropical Region stimulated the search for correlated molecular markers. Preliminary ITS1 sequence data suggested that differences do occur in samples of the AF complex throughout the whole region [31]. Furthermore, ITS1 sequences, with emphasis on samples from the Andean region, revealed one prevailing group in the south (Brazil, Argentina, Paraguay, Bolivia, and Peru), another in the northernmost areas (Mexico, Central America, Venezuela, and Colombia), and two other groups in the central and northern elevated regions of the Andes (Venezuela, Colombia, Ecuador, and Peru) [32]. Although the ITS1 sequences have been associated with geographic areas, their correlations with the distinct morphotypes have remained elusive, since they have not been identified as such in the analyses.

Apart from the recent significant increase in knowledge on cryptic species characterization within the AF complex, the lack of comprehensive diagnostic features has made accurate recognition of distinct taxa difficult. For example, in a microsatellite analysis, the samples of *A. fraterculus* were only partially associated with morphotypes [33], and in samples from Brazil throughout Mexico, the detected ITS1 sequences were associated only with geographic areas [32]. However, geographic location is not a reliable criterium for identification since distinct morphotypes may be found in sympatry as already described for some areas in Brazil [19,20,34]. Hence, there is urgency in re-evaluating genetic correlations within the AF complex by employing integrative approaches to specimens belonging to the same population samples. Herein, the proposal is to verify whether the morphotypes characterized by morphometric analysis have genetic support based on the sequences of the transcribed ribosomal nuclear spacer ITS1.

## 2. Materials and Methods

### 2.1. Biological Material

**Figure 1:**
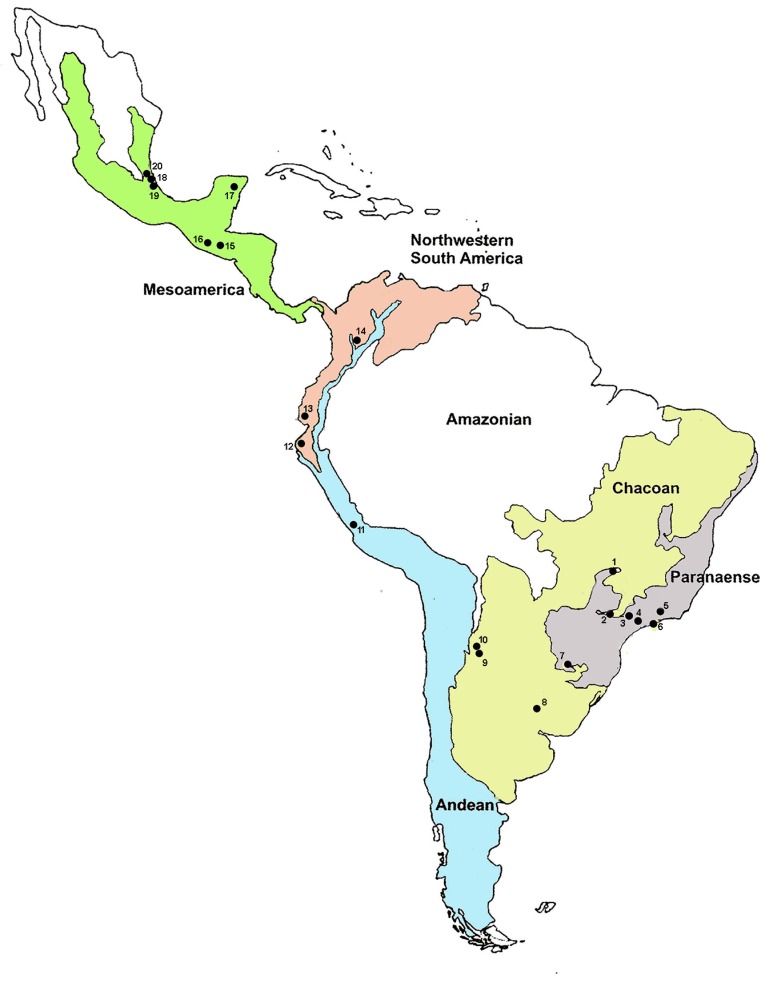
Collection sites of the *Anastrepha fraterculus* complex samples. Major biogeographical sub-areas in the Neotropical Region [28], showing the approximate locations of collection sites of the *A. fraterculus* samples: **Brazil:** 1. Uberlândia; 2. Botucatu; 3. Santa Isabel; 4. São Luís do Paraitinga; 5. Três Rios; 6. Ubatuba. **Argentina:** 7. Posadas; 8. Concordia; 9. Tucumán; 10. Horco Molle. **Peru:** 11. La Molina; 12. Piura. **Ecuador:** 13. Guayaquil. **Colombia:** 14. Ibagué. **Guatemala:** 15. Guatemala City. **Mexico:** 16. Tapachula; 17. Quintana Roo; 18. Teocelo; 19. Apazapan; 20. La Jicayana.

Specimens of the AF complex from 23 samples were obtained from 20 localities spread throughout the Neotropics: Mexico, Guatemala, Colombia, Ecuador, Peru, Argentina, and Brazil (Table 1 and Figure 1). Nineteen of these samples were derived from field-infested fruits and brought to the laboratory and kept until adult emergence, whereas two samples belonged to laboratory colonies, and two others were derived from McPhail traps. While all samples were screened for ITS1 sequences, 19 also underwent female wing-shape morphometry analysis. For the ITS1 analysis, adult females preserved in ethanol were used, except for those collected in Brazil, which emerged directly from the collected infested fruits. Adult flies from Brazil were identified using a combined analysis of biological characteristics [19,20], whereas flies from other countries were classified by morphometry, as previously proposed [27,28].

### 2.2. Analysis of ITS1 Sequences

DNA for the ITS1 analysis was extracted from 10 individuals of each sample collected in Brazil and from 5 individuals from each sample for the other localities. DNA was extracted from the isolated thorax of each individual in accordance with the Jowett protocol [36]. For PCR amplification, new primers based on the ITS1 sequence of *A. fraterculus* (*s.l*.) (GenBank #AY775552) were used, i.e., 18SF-TAACTCGCATTGATTAAGTCCC and 5.8SR-GATATGCGTTCAAATGTCGATG, which bind to the 3′ end of 18S and the 5′ end of 5.8S genes, respectively. PCR reactions consisted of one cycle (2 min at 94 °C), 31 cycles (30 s at 94 °C, 30 s at 60 °C, and 2 min at 68 °C) and a 5 min extension at 72 °C. Amplified products were electrophoresed in 0.8% agarose gel and visualized after ethidium bromide staining. Oneshot *Escherichia coli* cells were employed to generate five clones from each fragment by applying Topo TA Cloning (Invitrogen, Carlsbad, CA, USA). Plasmid DNA was extracted using the Perfectprep Plasmid Mini Kit (Eppendorf, Hamburg, Germany). The BigDye reaction kit in an ABI Prism Automatic Sequencer (Applied Biosystems, Foster City, CA, USA) was employed for sequencing at the Institute of Chemistry, University of São Paulo, São Paulo, Brazil.

Amplified fragments of around 900 bp contained complete ITS1 and partial sequences of the flanking genes. After comparison with the ITS1 sequences of *A. fraterculus* (*l.s.*) (GenBank #AY775552), the regions corresponding to 18S and 5.8S flanking genes were trimmed off. Amplification and sequencing of A or T homopolymeric regions are prone to the production of artifacts that are interpretable as either intra-individual polymorphisms or PCR slippage. Analysis of the ITS1 electropherograms of five clones from each sampled specimen with the Electropherogram Quality Analysis web tool [37] indicated the absence of intra-individual polymorphisms. Since 2 to 10 individuals were analyzed per sample, sequences differing, for example, in a homopolymeric region by only one extra nucleotide that was present in every individual of a given sample or in individuals from distinct samples were interpreted as a real nucleotide substitution and not as a PCR artifact. It would be highly unlikely that PCR errors had occurred in so many individuals at the same position in the sequences. The amplified ITS1 sequences were deposited in GenBank and are listed in Table 2.

Some of the samples analyzed herein (Table 1 and Table 2) were collected in geographic areas that overlap with samples analyzed by Sutton et al. [32], i.e., from Mexico/Guatemala, the lowlands of Peru/Ecuador, the highlands of Colombia, Peru, Bolivia, and south-eastern Brazil. For the sake of comparisons, the ITS1 sequences of the morphotypes identified herein were named accordingly as sequence types already described—types TI/TIa, TII, TIII(A,B,C), TIV—available in the GenBank [32]. Furthermore, three previously described sequences from Paraguay (HQ829865, HQ829868, HQ829876) were also analyzed [38]. The sequence set was aligned through Clustal Omega software [39], based on a previously proposed alignment [32], and genetic diversity was estimated by MEGA 6.0 [40]. Overall similarity among sequences was tested by UPGMA (unweighted pair-group method with arithmetic means) [41], the same methodology employed a priori to compare the ITS1 sequence types [32]. For the phylogenetic inferences the maximum likelihood (ML) [42] and fast minimum evolution (FME) provided at the NCBI site were also applied. The ML inference was made based on the best substitution model determined by the tool “Search the Best Model” implemented in Mega 6.0. According to the Akaike information criterion (corrected), the best model was the GTR (general time reversible) model. Regarding ML, the initial trees for the heuristic search were obtained automatically by applying the BioNJ method to a matrix of pairwise distances estimated using the maximum composite likelihood method [43] and then selecting the topology with the superior log likelihood value. The FME was based on pairwise distances inferred according to Jukes–Cantor methodology. Gamma distribution was used to model evolutionary rate differences among sites. The percentage of replicate trees in which the associated sequences clustered together was measured by bootstrap tests (1000 replicates). The analyses were conducted in MEGA 6.0.

### 2.3. Morphometric Assessment

Landmark-based geometric morphometry was applied to quantify and distinguish wing morphological features in 239 female specimens from 19 population samples of the AF complex (Table 1). Morphological studies were carried out using female wings since among the usual features employed in *Anastrepha* taxonomy (wings, ovipositor, mesonotum) it is the most appropriate to precisely recognize homologous landmarks [20,29].

For permanent preparations, the right-wings of 10–12 specimens per sample were removed from their bases, washed with distilled water, and later dehydrated in a gradual alcohol series (50%, 70%, 100%), with 20 min for each step. Then, they were placed in xylene for 2–3 min and mounted in a dorsal view between the slide and coverslip with Canada Balsam. Images of each wing were digitalized at an identical magnification with a high-resolution 5050 Olympus camera fitted to a Zeiss Stemi 508 stereomicroscope.

**Figure 2:**
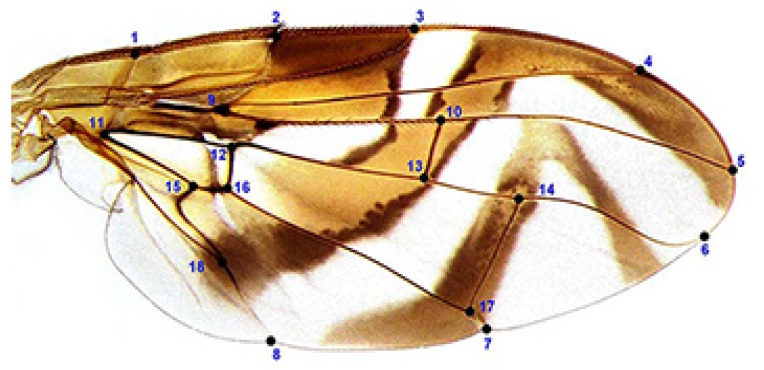
Female wing of a specimen of the AF complex. Right wing showing the 18 landmarks used for the geometric morphometric analysis.

The variations in female wing-shape were assessed from digital images by recording 18 homologous landmarks for each specimen using the TPS software package [44,45,46]. Landmarks were defined by the intersection or termination of wing veins, as previously described [29]: (1) junction of the humeral and costal veins; (2) subcostal break along the costal vein; (3) apex of vein R_1_; (4) apex of vein R_2+3_; (5) apex of vein R_4+5_; (6) apex of vein M; (7) apex of vein CuA_1_ on the posterior margin; (8) apex of vein CuA_2_ on the posterior margin; (9) basal bifurcation of R_2+3_ and R_4+5_; (10) junction of R_4+5_ and cross-vein r-m; (11) basal angle of cell bm; (12) junction of M and cross-vein dm-bm; (13) junction of M and cross-vein r-m; (14) junction of M and cross-vein dm-cu; (15) junction of CuA_1_ and Cu_2_; (16) junction of CuA_1_ and cross-vein bm-cu; (17) junction of CuA1 and dm-cu; and (18) junction of A and Cu_2_ (=apex of cell bcu) (Figure 2).

A generalized Procrustes analysis using the MorphoJ program was conducted by overlapping landmark configurations that remove variations caused by differences in translation, orientation, and size into a common coordinate system [47]. A Canonical variate analysis was performed with the matrix of Procrustes coordinates for 19 population samples. A cluster analysis of phenotypic similarities using the matrix of squared Mahalanobis distances (SMD) involving pairwise comparisons by the UPGMA method (unweighted pair-group method with arithmetic means) was implemented in the Statistica software program (StatSoft, Inc., Tulsa, OK, USA, 2006). A morphotype centroid-size analysis was done with a Kruskal–Wallis test, followed by Dunn’s multiple comparisons with Bonferroni correction through the PMCMR package in the program R [48]. Reference samples and permanently mounted slides were deposited at the Entomological Collection of the Instituto de Ecología A.C., Xalapa, México, and the wing preparations from the Brazilian samples were deposited in the Laboratory of Evolutionary Studies on Fruit Flies, Department of Genetics and Evolutionary Biology, Institute of Bioscience, University of São Paulo, São Paulo, Brazil.

## 3. Results

### 3.1. Analyses of ITS1 Sequences

**Figure 3:**
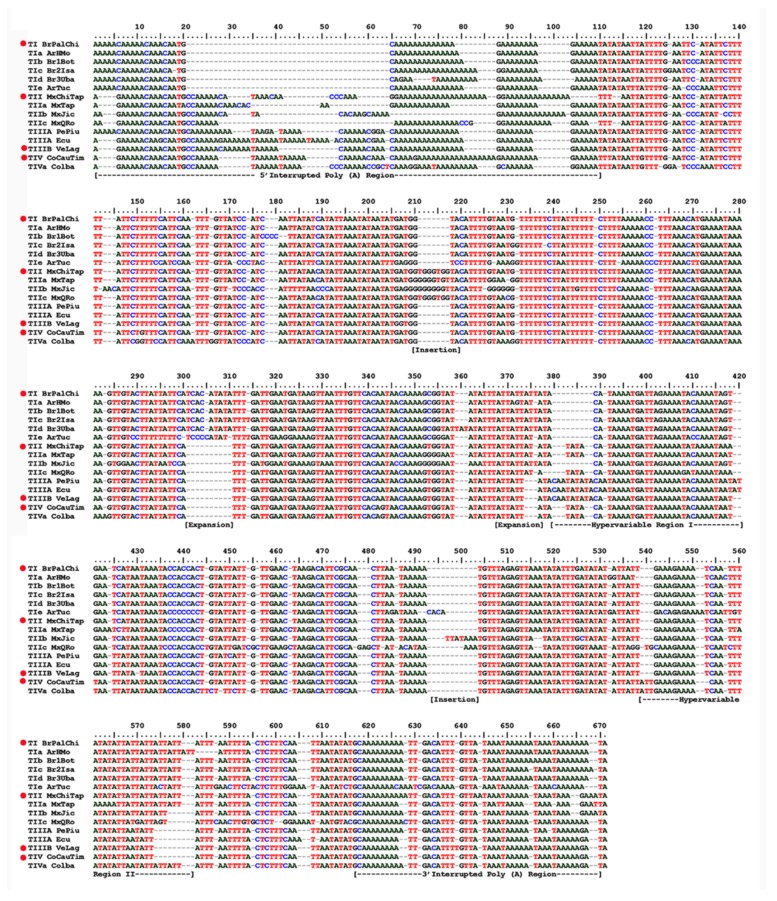
Alignment of representative ITS1 sequences of the AF complex. Sequences are identified as TI to TIV, and the variants are labelled as TIa to TIe, and TIVa. Underneath the alignment, special landmarks of the sequences are indicated. The red dots indicate Sutton et al.’s sequences [32].

The 32 ITS1 sequences obtained varied from 530 to 583 nucleotide (nt) positions, were rich in AT (ca. 70–84%),and showed complex structures. As described in detail for other samples of the AF complex [32], the sequences had poly (A)/poly (T) portions, highly variable regions interspersed with conserved subsequences, and scattered SNPs (single nucleotide polymorphism). Although most samples had a single sequence type, in some, 2 to 3 sequences were detected with 1 or 2 nucleotide substitutions. Since this represents a similarity of 99% or more, it was assumed that the sequences were alike, and therefore, the sample was represented by a single sequence, i.e., the predominant one (Table 3). Classification of the representative sequences obtained from distinct AF morphotypes compared with representative sequence types found previously [32] are described below and shown in Figure 3. However, inconsistencies were found in data related to the collection sites, sequence types, and the nucleotide sequences in the GenBank. For example, in [32] (Table 2), collections 43–44 from Colombia (San Andrés Island) are listed as having the TI/TIa sequence type, whereas in the text [32] (p. 186), they are stated as being type TII. A comparative analysis of the sequences deposited in the GenBank showed they have 99% similarity with sequence type TII, which is found in Mexico, Central America, and Venezuela. Another case involving sequences from Colombia (collections 17–20, 25, 40, 41) is listed as being type TIIIC. An analysis of sequences from GenBank indicated the presence of SNPs and one unique repeat ATT, as described in [32] (p. 188), thereby proving that they are of type TIV. Thus, the types of ITS1 sequences obtained were determined by comparisons with a set of 29 sequences available in GenBank for which the respective accession codes could be unambiguously correlated with their collection sites (localities and altitude) [32]. These sequences are listed in Table 2.

### 3.2. Characterization of the ITS1 Sequence Types

The representative ITS1 sequences obtained were aligned with previously distinguished representative sequence types—types TI, TIa, TII, TIIIABC, TIV [32]—from sites overlapping those of the present analysis (Figure 3). ITS1 sequences have an interrupted poly(A) region and bases 1–109 and 618–669 (the numbers refer to the nucleotide positions shown in the alignment of Figure 3), respectively, at their 5′ and 3′ ends. In the intervening regions, there are two hypervariable subregions, HVRI and HVRII, with bases 380–420 and 539–582, respectively, interspersed with conserved subsequences and scattered SNPs (single nucleotide polymorphism). However, as the alignment of the 5´Interrupted Poly (A) Region (bases 1–109) is clearly problematic due to the high variability among samples, these were removed in the characterization of the ITS1 sequence of the distinct morphotypes.

#### 3.2.1. Sequence Type TI

This type, encountered in samples from south-eastern Brazil, Paraguay, Bolivia, and the highlands of Peru, has a unique TCACATATA expansion (bases 301–310), an ATT extension (bases 368–376), and SNPs at bases 353, 403, and 417. A variant of this sequence, TIa, besides the TI markers, has a unique ATT extension (bases 580–582) inside Highly Variable Region II in samples from Peru (Pe96VRreg, Pe63AyaHua) and Bolivia (BoSCr). TIa was also found in the ArHmol sample from Argentina. Other samples of Brazilian morphotypes had the basic TI markers but showed differences characterizing other TI variants. The sequences from the Brazilian-1 morphotype had characteristic CCC (bases 129–131) and CCCC (bases 177–180) expansions, as well as a few other SNPs; sequences of Brazilian-2 morphotype had an additional G at positions 125 and 232, an additional A at position 668, and other nucleotide substitutions. The sequences found in the three samples of Brazilian-3 morphotype had a unique TAT extension at positions 359–361 and other SNPs. Hence, the sequences were considered new variants of TI associated specifically with the three Brazilian morphotypes and were named TIb (Brazilian-1), TIc (Brazilian-2), and TId (Brazilian-3). The sequence from Tucumán (ArTuc, Argentina) was considered a variant of TI but was distinct from the other variants (TIa to TId) due to its unique extensions, (T_9_ (bases 291–298), C_4_ (bases 302–305), CACA (bases 493–496), and GGAAA (bases 602–606)), as well as several SNPs, and as such, it was named TIe. Genetic distances between these sequence types were low, the highest being those of ArHmol (mean of 0.014) and ArTuc (mean of 0.142) relative to the other samples (Table S1).

#### 3.2.2. Sequence Type TII

These sequences are characterized by a putative reduction of TATAT to TTT (bases 110–114), a unique expansion of TGGGTGG (bases 211–217), another expansion of (T)_6_ to (T)_7_ at position 249, and (ATT)_n_ (n = 4 and 5) repeats (bases 565–579), as well as SNPs at bases 137, 190, 410, and 669. Sequence type TII was found in samples from Mexico, Central America, the lowlands of Venezuela, and San Andrés Island (Colombia). However, samples from the three localities from Mexico showed relevant differences in SNPs and unique insertions, namely MxJic, AAC (bases 143–145), GGGGG (bases 227–230), TTATAAA (bases 497–503); MxQro, TTT (bases 110–112), GAG (bases 478–480), AAA (bases 501–503), TG (bases 540–541); and MxTap, A_6_ (bases 560–565), and GGGGA (bases 353–356). The average intragroup genetic distance between sequences was 0.039 (Table S2). Although the Mexican morphotype is characterized by sequence type TII, the high level of divergence in certain populations, due to the presence of numerous unique markers indicates the presence of variants, TIIa (MxJic), TIIb (MxQro), and TIIc (MxTap).

#### 3.2.3. Sequence Types TIII A, B, C

Although mutually very similar, TIII A was characterized by an expansion in the HVRI CAATATATA (bases 380–388) by insertions of an A (bases 391 and 569) and an AT (positions 419–420) and possible A expansions at base 605. This sequence type was found in the lowlands of Peru and Ecuador (<900 m) but showed little divergence between the sequences PeCaj, PePiu, and PeLmo. The average distance between TIIIA sequences was 0.002 (Table S3); consequently, the sequence type TIIIA characterized the Peruvian morphotype.

#### 3.2.4. Sequence Type TIV

This type has unique SNPs at positions 119, 151, 343, and 421 and a unique repeat expansion (ATT)_n_ (n = 2 and 3) at positions 539–541. Every sequence from the highlands of Colombia (>1500 m) was type IV, and the average distance between samples was 0.006 (Table S4). The sample from Ibagué (CoIba) obtained differed by possessing several additional SNPs other than the four common ones of sequence type TIV, as well as the addition of G, T, or A at bases 125, 131, 137,139,162, 166, 232, 243, and 445. Furthermore, the CoIba sequence also showed the unique repeat expansion (ATT)_3_ characteristic of sequence type TIV. The data indicate that Andean morphotypes are characterized by having sequence type TIV, with at least one variant, CoIba, which could be named TIVa.

### 3.3. Relationships of Sequence Types among Samples of the AF Complex

**Figure 4:**
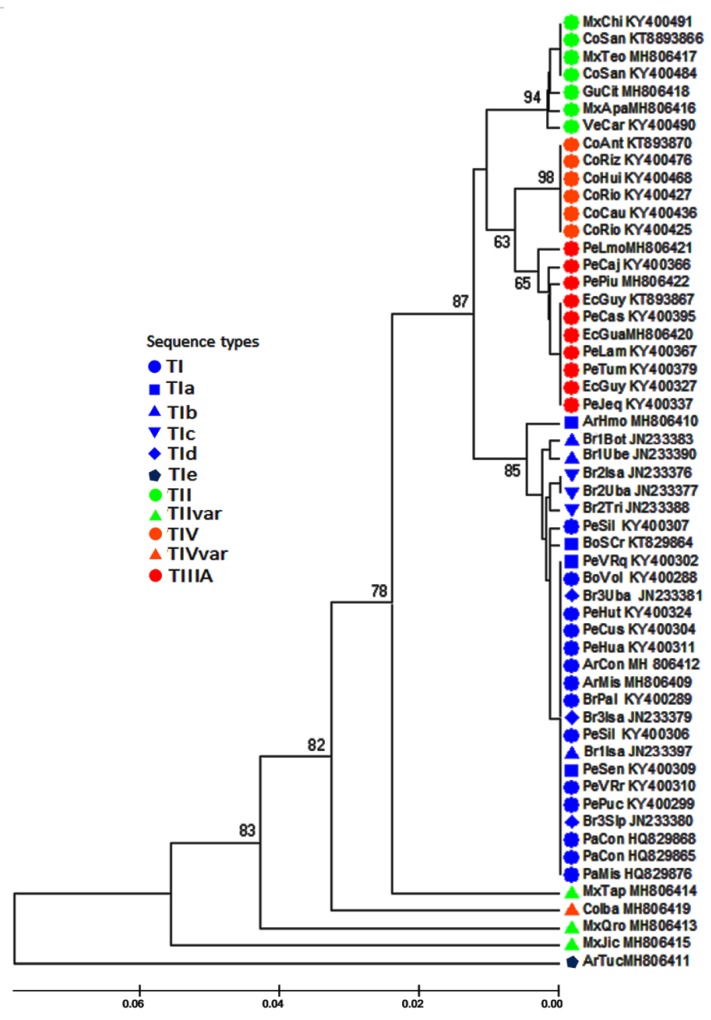
Overall similarity of the ITS1 sequences of the AF complex. The similarity of the sequences was inferred using the UPGMA (unweighted pair-group method with arithmetic means) method. The percentage of replicate trees in which the associated taxa clustered together in the bootstrap test (1000 replicates) are shown next to the branches. The evolutionary distances were computed using the maximum composite likelihood method and are in units of the number of base substitutions per site. The rate variation among sites was modeled with a gamma distribution (shape parameter =1). All positions containing gaps and missing data were eliminated.

To achieve an improved comparison between the sequences obtained with those previously described [32], the similarities among the entire set of 55 ITS1 sequences (Table 2) were inferred by a UPGMA methodology. The results are shown in Figure 4. UPGMA revealed four main clusters and placed four sequences as single terminal branches: one from Argentina (ArTuc), one from Colombia (CoIba), and three from Mexico (MxTap, MxQro, MxJic). Each of the main cluster aggregates’ specific sequence types corresponded to a distinct morphotype of the AF complex: One cluster with sequence types TIV and TIVa corresponded to the Andean morphotype, another with the TIIIA sequence corresponded to the Peruvian morphotype, and one cluster with sequence type TII was found in the Mexican morphotype, as well as a large cluster with sequence type TI and its variants. The latter was found in samples from the highlands of Peru and Bolivia as well as Paraguay, Argentina, and south and south-eastern Brazil. This cluster was subdivided into a branch with the ArHmol sequence from Argentina (type TIa), a sub-cluster from Brazil with Brazilian-1 morphotype (TIb), a branch from Peru with the sequence Pe64AyaPon (type TI), another sub-cluster from Brazil comprising morphotype Brazilian-2 with its sequence (TIc), and a larger sub-cluster comprising sequence types TI and TIa from Peru, Bolivia, and Paraguay, sequences of morphotype Brazilian-3 from Brazil (TId), and sequences from Argentina. The sequence Br09ParCha (type TI) included in this sub-cluster is the only one available in GenBank among all the studied samples from Brazil [32].

**Figure 5:**
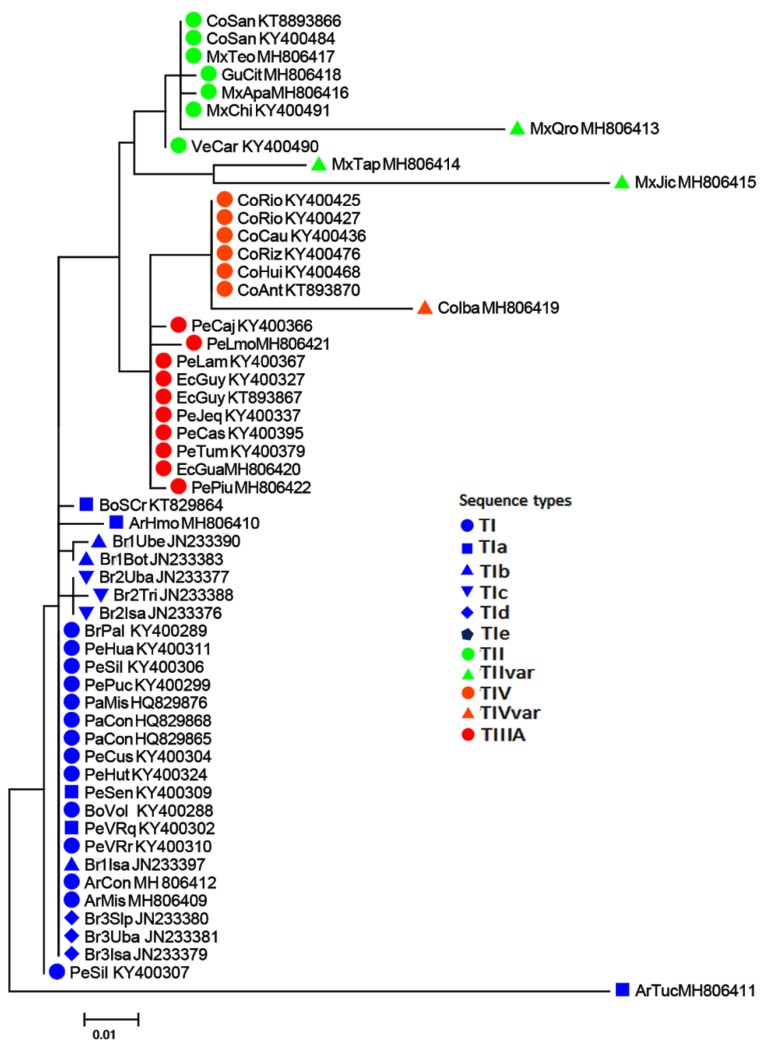
Phylogenetic relationships of the ITS1 sequences of the AF complex. The evolutionary history was inferred by using the maximum likelihood method based on the general time reversible model. The tree with the highest log likelihood is shown. Initial tree(s) for the heuristic search were obtained by applying the BioNJ method to a matrix of pairwise distances estimated using the maximum composite likelihood (MCL) approach. A discrete gamma distribution was used to model evolutionary rate differences among sites. The numbers on the branches are bootstrap values >50% (1000 replications). All positions containing gaps and missing data were eliminated.

Additionally, the genetic relationships among the sequences were inferred by a maximum likelihood phylogenetic analysis by employing the entire set of ITS1 sequences. The ML tree showed that the sequences were also grouped in four clades, each of which congregated the same set of sequences as those included in the clusters of the UPGMA analysis (Figure 5). The clade with samples from the Mexican morphotype was the most basal. There was also a clade with sequences from the Peruvian morphotype, one with sequences of the Andean morphotype, and the last one with sequences of the three Brazilian morphotypes and the sequences TI/TIa from Peru, Bolivia, Paraguay, and Argentina. Within this clade, the sequences of the Brazilian-1 and Brazilian-2 samples were separated into distinct, although internal, sub-clades, while the sequences of Brazilian-3 and all the other sequences were in another sub-clade. The ArTuc (Argentina) sequence and two sequences from Mexico (MxTap, MxJIc) were isolated in basal terminal branches, similar to the UPGMA dendrogram, but the sequences MxQro and CoIba isolated as basal terminal branches in the UPGMA were included within the clades of the Mexican and Andean morphotypes, respectively, in the ML analysis. The second phylogenetic inference was made by the fast minimum evolution methodology, and the topology was very similar to that found by the ML inference. The main distinction is that the three Brazilian sub-clades were more distinctly separated from each other and from the other sequences, and two of the Mexican morphotypes (MxTap; MxJic) were still isolated basal terminal branches (Figure S1).

### 3.4. Morphological Relationships

A morphometric analysis of the female wing shapes of the 19 population samples showed the presence of at least six morphotypes within the AF complex. An overall examination indicated that all canonical variates (CV) were statistically significant (Wilks’ Lambda: 0.00251; F _(155,1009)_ = 15.327; *p* < 0.0001). The first two accounted for 75.7% of the group differentiation (CV1 = 55.7% and CV2 = 20%), whereas the remaining CV3, CV4, and CV5 contributed 11%, 9.8%, and 3.7%, respectively.

**Figure 6:**
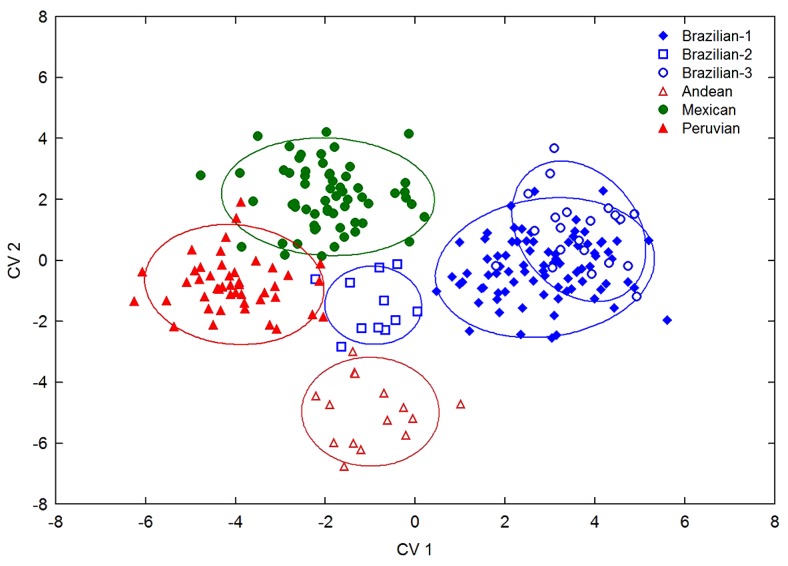
Canonical variates analysis (CVA) of wing shape. Plot of the first two canonical variates that resulted from the analysis of wing shapes from 19 population samples. The CVA analysis was used to test the hypothetical groups by morphotype. Confidence ellipses 95%.

A comparison of morphological distances among the three Brazilian groups exhibited high contrast, with the greatest similarity reported between Brazilian-1/Brazilian-3 (SMD = 7.17), whereas for Brazilian-1/Brazilian-2 (SMD = 45.9) and Brazilian-2/Brazilian-3 (SMD = 49.2), the values were up to six times lower. In other geographical regions in the Americas, a close similarity was also noted for the wing-shape between Peruvian/Mexican (SMD = 9.18), as compared with the low similarity between Mexican/Andean morphotypes (SMD = 55). Meanwhile, minor similarities in wing shape were found between the Brazilian-3/Andean (SMD = 70.4) and Brazilian-3/Peruvian morphotypes (SMD = 74.2) (Figure 6).

**Figure 7:**
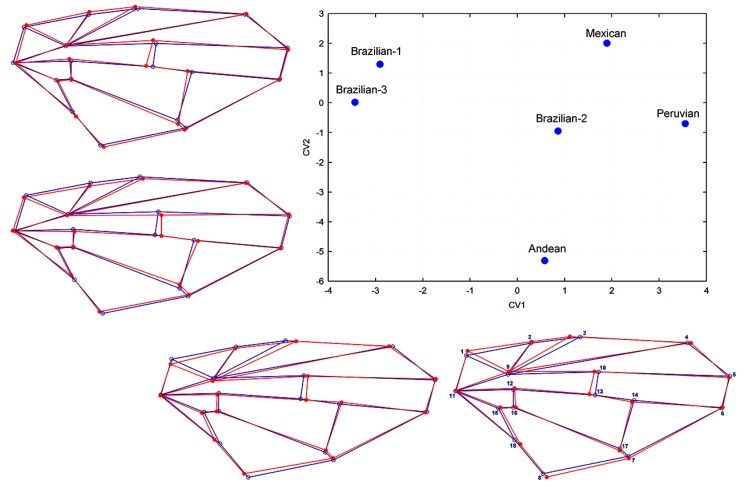
Centroid function and shape deformation of female wing shapes. Graph showing the centroid functions of the first two canonical variates based on 18 landmarks of wings resulting from comparisons among the six AF complex morphotypes. Wireframes show shape deformations (red line) from the configuration consensus (blue line) for each positive and negative (scale factor 10) extreme canonical variate.

Partial-warp scores captured the shape variation to visualize the major trends associated with hypothetical changes found in the samples. These shifts in wing shape are depicted by an outline drawing and plot of the two first canonical variates by morphotype (Figure 7).

**Figure 8:**
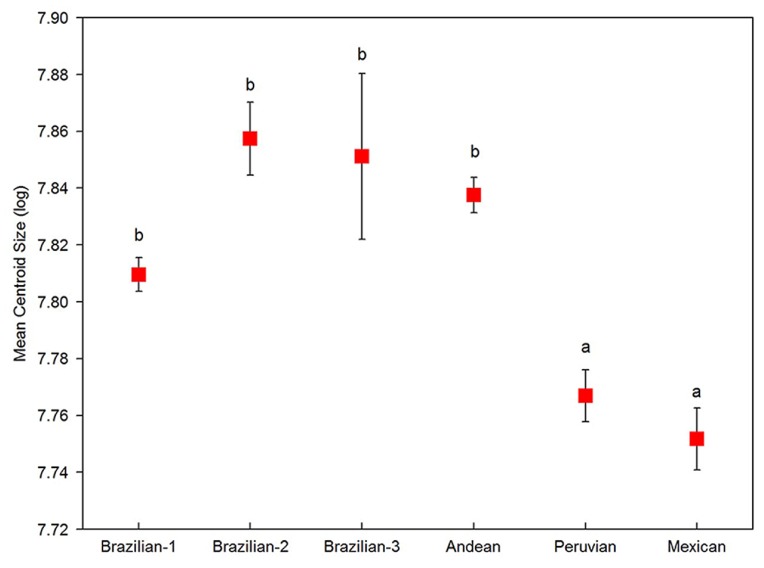
Centroid size of female wings. The mean centroid size (±1 SE) of female wings by morphotype of the *A. fraterculus* complex. Different letters mean that values have statistical significance based on Dunn’s test (*p* < 0.05).

The centroid size used to measure geometrical dimensions was compared among morphotypes resulting in a significant differentiation of groups (Kruskal–Wallis: χ^2^ = 50.93, *p* < 0.0001), showing that the Mexican and Peruvian groups possess smaller wing sizes than all the other morphotypes (Dunn test in all *p* < 0.010 comparisons) (Figure 8). Furthermore, the model designed to distinguish among morphotypes was confirmed by the classification of the 239 individuals examined, as 99.2% were classified properly into expected clusters, proving the high reliability of the predictive model to distinguish them based on natural groups (Table 4).

### 3.5. Wings and ITS1 Data Relationships

**Figure 9:**
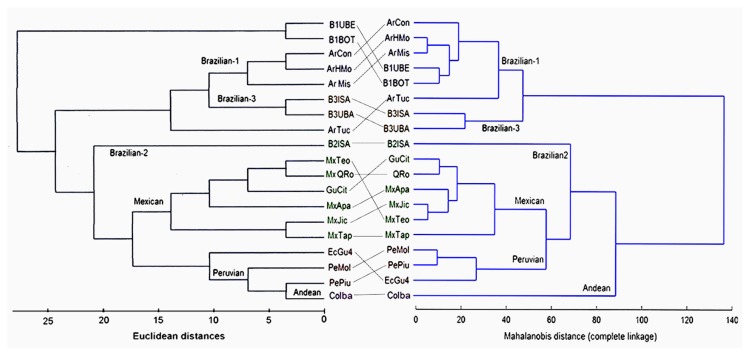
Dendrograms of the female wing shape and ITS1 sequences: (Right) UPGMA clustering of morphometric data based on the wing shapes of females from 19 population samples of the AF complex. (Left) UPGMA clustering of ITS1 sequences of the same populations. The names in the branches correspond to the morphotype classification.

A comparison between genetic and morphological data was conducted on the 19 population samples analyzed by the two methods (Figure 9).

Clustering analyses revealed an aggregation into two widely discrete sets of samples, the first one consisting of four Argentinian populations (ArCon, ArHmo, ArMis, ArTuc), along with two Brazilian ones (B1Bot, B1Ube)—all of them recognized as the Brazilian-1 morphotype [28] (sensu Hernández-Ortiz et al.)—and another one including samples B3Isa and B3Uba, which belong to the Brazilian-3 morphotype [19] (sensu Selivon et al.). Although somewhat divergent in topology, the clustering of ITS1 sequences produced a similar grouping, which separated the samples of Brazilian-1, Brazilian-3, and Argentina from all the other remaining samples. The samples from Argentina exhibited higher similarity with those of Brazilian-3 than those of Brazilian-1.

The second group consisted of all remaining populations clustered into four clusters, each of them identifying a distinct morphotype: (1) Andean, represented by a Colombian highland sample (CoIba) showing the largest divergence in wing shape, even among the nearest geographical samples, e.g., Peru and Ecuador; (2) Brazilian-2, depicted separately by only one Brazilian population (B2Isa); (3) Mexican, including all samples from Mexico and Guatemala (GuCit, MxQro, MxApa, MxJic, MxTeo, MxTap); and (4) Peruvian, composed of the Peruvian and Ecuadorian Pacific lowland samples (PeMol, PePiu, EcGu4). The clustering of ITS1 sequences from these samples yielded a highly similar dendrogram that separates the Brazilian-2 (Br2Isa) morphotype sequence from two other clades: one grouping the Mexican morphotype sequences and another one consisting of the three Peruvian morphotype samples, along with the Colombian (CoIba) sample from the Andean morphotype, which was not differentiated, as shown in the morphotype dendrogram.

## 4. Discussion

From a morphological point of view, the AF complex comprises at least eight morphotypes. The genetic divergence correlates with morphological differences for six of these morphotypes, as described in the present analysis, strongly confirming the existence of various taxonomic species within the AF complex. A previous analysis of ITS1 in samples of the AF complex revealed high distinctiveness throughout the Neotropical Region [31,32,38]. However, the lack of morphological characterization in these studies precluded the association of the ITS1 sequence types with the various morphotypes. This report presents results from the correlated analysis of morphometry and ITS1 data in specimens from the same populations.

### 4.1. ITS1 as a Molecular Marker

This molecular marker has been used in the genetic analysis of very closely related taxa for which a recent evolutionary history is assumed [49]. The multiple copies of the ITS1 present in a genome might complicate a genetic analysis if differences do exist among them, but the cloning of ITS1 fragments in the present analysis detected no intra-individual variations. As the intra-genome similarity of ITS1 copies has been reported for several organisms, molecular-drive mechanisms are assumed to explain the observed intra-individual homogeneities [50,51].

Differences in the fragment size were not detected in a previous ITS1 analysis of Argentinian samples [52]. If only the size of the amplified fragment had been considered herein, we would also have found no relevant differences between the samples, but the scenario is quite different when the sequences of such fragments are considered.

According to the data, the overall nucleotide variability of ITS1 sequences is generally low, varying from 0.006 (Brazilian-3) to 0.039 (Mexican). This is surprising, since non-coding regions have a tendency to accumulate mutations faster than coding sequences [53,54]. Regardless of the molecular marker employed, low genetic variability in *Anastrepha* samples has often been observed. Some hypotheses have been proposed to explain this phenomenon although the underlying cause(s) remain unknown [11,12,55,56,57,58]. The low genetic distance estimated through ITS1 sequences between AF complex morphotypes could be attributed to a possible recent evolutionary divergence of the complex [11,20]. Nevertheless, this does not obscure distinction among the morphotypes, since peculiarities in ITS1 sequence structures in each of the six studied morphotypes really do exist.

### 4.2. ITS1 and Morphotype Relationships

**Figure 10:**
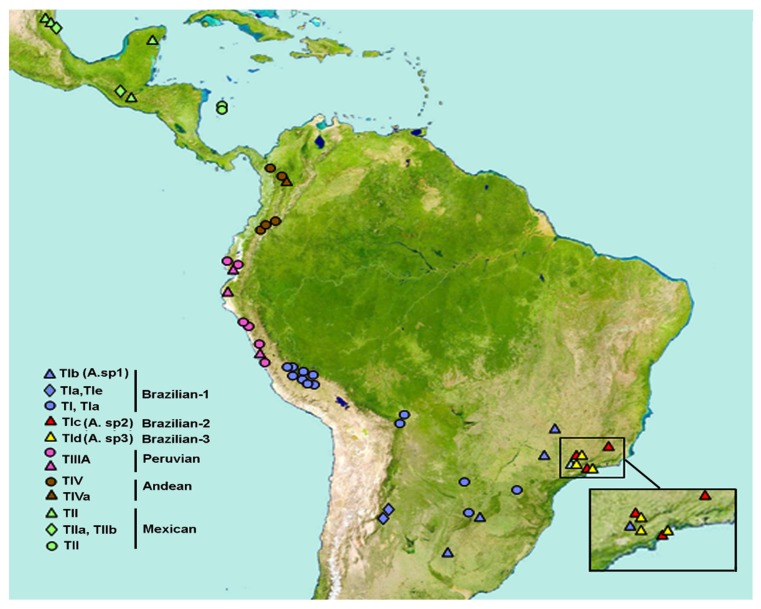
Approximate geographical location of *Anastrepha fraterculus* morphotypes and related ITS1 sequence types identified herein.

Sequence type TI and the variant TIa, detected in an extensive area throughout south/south-eastern Brazil, Argentina, Bolivia, the highlands of Peru (Cusco region), and in Paraguay, were putatively identifiable as *Anastrepha* sp.1 aff. *fraterculus* (Brazilian-1 morphotype) [32]. However, the present data showed quite different results. Although all the samples from the highlands of Peru and Bolivia do have sequence types TI or TIa, those from Paraguay were found to be closely related to Brazilian-3, hence corroborating previous data [38]. Moreover, the Brazilian morphotypes [32] collected from the inland plateau of south/south-eastern Brazil presented sequence types that were similar, but not identical to, TI or TIa. Notably, no shared ITS1 sequences were detected in sympatric zones, e.g., the valley of the “Paraiba do Sul” river, where the three Brazilian morphotypes coexist (samples Br1Isa, Br2Isa, Br3Isa), nor in the coastal region of south-eastern Brazil, where *A*. sp.2 and *A*. sp.3 coexist (samples Br2Uba, Br3Uba) [19,20,34]. Distinct sequences characterize the other morphotypes: the Mexican morphotype is characterized by sequence type TII but with variants in three samples from Mexico; the Peruvian morphotype (lowlands of Peru and Ecuador) by sequence type TIIIA; and the Andean morphotype (highlands of Colombia) by sequence type TIV and a variant TIVa. Figure 10 summarizes information on the geographical occurrence of each morphotype associated with ITS1 sequences from Sutton et al. [32] and the present data. The specificity of the ITS1 sequences in each of these morphotypes is not surprising when considering the geographic distances between their occurrence zones. Although contact between the different morphotypes could have happened through anthropogenic actions, an important mechanism of fruit fly dispersion [15], the present results do not provide evidence of this.

In Figure 10, symbols of different colors indicate the ITS1 sequence types found for each morphotype. The circles indicate the ITS1 sequence types described by Sutton et al. [32] but with putative identification of morphotypes based on data from the present study. The inset shows the sympatric zones in south-eastern Brazil where the Brazilian morphotypes coexist.

Morphotype distinctiveness was in line with genetic differences revealed by the ITS-1 analysis, which, in turn, complied with the mutual reproductive incompatibility encountered in experimental crosses carried out with almost all of the morphotypes [20,30]. Variations observed in cytogenetic and genetic analyses of some populations from Argentina were interpreted as polymorphisms, and mating compatibility between some populations led to the assumption of the existence of a single regional taxon [59,60,61,62]. However, reproductive isolation, or conversely, compatibility, may not be the sole criterium to define species boundaries [63]. The present ITS1 and morphometric data, as well as previous linear morphometric results [27,28], revealed differences in Argentinian samples of such a magnitude that the presence of distinct entities may not be ruled out.

Morphological variation among samples from Mexico has already been described [27,28,29]. The present results additionally confirm these morphological variations and reveal a partial congruence with genetic data found in the ITS1 sequences. The data suggest that further analysis is necessary to clarify the real meaning of such differences.

The existence of various biological entities within this species complex may be related to the wide continental distribution encompassing the diverse ecological conditions and geographic areas where they occur. Supposedly, the geographic distance could prevail in the maintenance of divergence. Nevertheless, reproductive isolation laboratory tests between distant geographic samples [64,65,66,67] suggested that the isolation between morphotypes has been established in the past with enough time for the appearance of reproductive barriers.

Since the Brazilian morphotypes, as a whole, have been well characterized through integrated analyses of several biological parameters, including reproductive isolation assays, they have been described as different species in previous studies (*A*. sp. 1, *A*. sp. 2 and *A*. sp. 3) [19,20,67]. A genetic distance of 0.128 was estimated by the isozymic analysis of 19 loci between Brazilian morphotypes. In contrast, a detailed analysis of 20 gene sequences, all subjected to strong purifying selection, registered the absence of genetic divergence among 16 samples of the nominal *Anastrepha fraterculus* from Brazil [68]. This is remarkable, since more than one species of the AF complex occurs in some of the sampled localities [19,20], and at least four isozyme diagnostic loci exist between *A*. sp.1 and *A*.sp.2 [20]. Thus, the absence of genetic divergence observed in [68] aligns with the low genetic divergence (0.004) between the ITS1 of the Brazilian morphotypes, which could contradict the recognition of morphotypes as units with distinct evolutionary pathways. However, as the authors themselves pointed out [68], their analysis involved limited parts of the genome, thereby limiting the detection of differentiation among the samples.

### 4.3. Scenarios of Divergences Within the AF Complex

No areas of sympatry have been described for the AF complex, except for the Brazilian morphotypes, and the knowledge of their host fruits is scarce. Crosses between specimens from south Brazil (*A*. sp.1 according to the authors) and flies from Argentina were found to be fertile [69], but it must be noted that in south Brazil, *A*. sp.3 is found in sympatry with *A*. sp.1 [19,70,71]. Thus, based on the existing information, it is reasonable to assume that divergence has been established in allopatric scenarios. It must be noted that in the present analysis, no evidence of hybridization was found among the Brazilian morphotypes, even among samples collected in zones of sympatry, a prerequisite for the occurrence of hybridization. This is contrary to the presumed evidence of hybridization inferred through an analysis of a sample from a laboratory colony of *A. fraterculus* from south-eastern Brazil, which led to the proposition that homoploid hybrid speciation would be a prevalent process [72].

Considering the simplest model of ecological speciation, the occupation of different ecological niches or different geographic environments could result in reproductive isolation [73]. It is recognized that allopatry and secondary gene flow can be determinants in the speciation processes, as was hypothesized for host plant shifts and host race formation in *Rhagoletis.* It was postulated that initial allopatry would furnish the genetic architecture conducive to a sympatric host shift for *Rhagoletis pomonella* divergence [74]. One may assume that such processes are involved in the evolutionary history of the Brazilian morphotypes, which are found in both allopatry and sympatry, presenting differences in the host fruit usage and reproductive incompatibilities ensured by pre- and post-mating barriers [19,20,67]. The lack of more detailed information on the different morphotypes of the AF complex, such as geographic distribution and host fruit usage, make the reconstruction of its evolutionary history premature. Still, it is reasonable to suppose a dynamic scenario in which allopatry and the exploration of different ecological niches act as driving forces in the divergence process. A dynamical analysis based in “species network” performed by generalized logical formalism methodology involving the three Brazilian morphotypes, their host fruits, and geographic distribution, revealed an unidirectional incompatibility between *A*. sp.1 and *A*. sp.2, in addition to an environmental factor related to altitude, that are involved in the distribution pattern currently observed [34]. The detailed knowledge of factors involved in the relationships between species as obtained by the dynamic analysis associated with results of integrative studies would be critical to reconstruct the scenario of divergence within the AF complex.

## 5. Conclusions

The present report describes the results of an integrated genetic and morphologic comprehensive analysis of individuals belonging to the same population samples of the AF complex morphotypes. The morphometrical analysis clearly identified six morphotypes in the samples out of the eight described for the AF complex: Brazilian-1, Brazilian-2, Brazilian-3, Peruvian, Andean, and Mexican. The analysis of ITS-1 detected specific sequences for each morphotype, thus showing a strong congruence between genetic and morphological data. Moreover, the morphological and genetic variations revealed in the Argentinian and Mexican samples indicate that further research should be done to better understand its true meaning. Overall, the data clearly confirm that morphotypes are distinct biological species bearing genetic differences, and potential tools for recognizing the taxa of the AF complex are described, which may be useful for academic and applied purposes.

## Figures and Tables

**Table 1:** Biogeographical variables of AF complex collection sites.

Country	Provinces *	Locality	Map	Coordinates	Host Fruit
Brazil	Parana	Uberlândia	1	18°56′46″ S, 48°13′55″ W	Guava (*Psidium guajava*)
		Botucatu	2	22°56′18″ S, 48°18′25″ W	Guava
		Santa Isabel **	3	23°19′00″ S, 46°13′25″ W	Guava; Orange (*Citrus sp*.)
	Parana Florest	Três Rios **	5	22°07′21″ S, 45°41′52″ W	Guava; Orange
		São Luis do Paraitinga **	4	23°13′24″ S, 45°18′47″ W	Guava
	Atlantic	Ubatuba	6	22°46′24″ S, 48°18′25″ W	Tropical almond (*Teminalia cattapa*)
Argentina	Parana	Misiones	7	27°23′59″ S, 55°56′01″ W	Guava
	Pampeana	Concordia	8	31°23′13″ S, 58°01′12″ W	Guava
	Chaco	Tucumán	9	27°02′18″ S, 65°19′13″ W	Guava
		Horco Molle	10	26°46′37″ S, 65°19′49″ W	Guava
Peru	Desert	La Molina	11	12°14′15″ S, 76°31′50″ W	Lab colony (*Annona cherimola*)
	Ecuadorian	Piura	12	07°40′23″ S, 79°12′40″ W	Guava
Ecuador	West Ecuadorian	Guayaquil	13	02°2′13″ S, 79°53′50″ W	Guava
Colombia	Paramo	Ibagué	14	04°26′11″ N, 5°11′29″ W	Lab colony (*Coffea arabica*)
Guatemala	High Altitude Chiapas	Guatemala City	15	14°36′51″ N, 0°32′22″ W	Guava
Mexico	Pacific Low Land	Tapachula	16	14°53′47″ N, 2°10′30″ W	Guava; Loquat (*Eriobotrya japonica*)
	Yucatan	Quintana Roo	17	19°37′39″ N, 8°38′56″ W	McPhail trap
	Veracruz	Teocelo	18	19°23′14″ N, 6°57′23″ W	Guava
		Apazapan	19	19°17′00″ N, 6°36′59″ W	McPhail trap
		La Jicayana	20	19°21′44″ N, 6°39′23″ W	Guava

(*) Based on [35]; (**) Samples not included in the morphometric analysis.

**Table 2:** Country, locality, code, sample and NCBI accession of ITS1 sequences of the AF complex.

Country	Locality	Code	Sample	NCBI Accession *
Brazil	Palmas, PR	BrPal	9	KY400289
	Uberlândia, MG	BrUbe	1	JN223390
	Botucatú, SP	BrBot	2	JN223383
	Santa Isabel, SP	BrIsa	3	JN223376; JN1223379; JN223385
	S. L. Paraitinga, SP	BrSlp	4	JN223382
	Três Rios, RJ	BrTri	5	JN223378
	Ubatuba, SP	BrUba	6	JN223377; JN223380
Argentina	Misiones	ArMis	7	MH806409
	Concordia	ArCon	8	MH806412
	Tucumán	ArTuc	9	MH806411
	Horco Molle	ArHmo	10	MH806410
Bolivia	Santa Cruz	BoSCr	3	KT829864
	Los Volcanes	BoVol	4	KT400288
Peru (highlands)	Huatatas	PeHut	63	KY400324
	Pucahuasi	PePuc	65	KY400299
	Huanta	PeHua	66	KY400311
	Cusco	PeCus	68	KY400304
	Sillacancha	PeSil	70	KY400306; KY400307
	VRAE Qanan	PeVRq	95	KY400302
	SENASA	PeSen	96	KY400309
	VRAERegion	PeVRr	97	KY400310
Peru (lowlands)	La Molina	PeLmo	11	MH806421
	Piura	PePiu	12	MH806422
	Casma	PeCas	61	KY400395
	Cajamarca	PeCaj	67	KY400366
	Lambayeque	PeLam	80	KY400367
	Jequetepeque	PeJeq	83	KY400337
	Tumbes	PeTum	92	KY400379
Ecuador	Guayas	EcGuy	49	KY400327; KT893867
	Guayaquil	EcGua	13	MH806420
Colombia (island) (Highlands)	San Andres	CoSan	43; 44	KY400484; KT893866
	Ibagué	CoIba	14	MH806419
	Rionegro	CoAnt	17	KT893870
	CoRio	18;19	KY400427; KY400425
	CoCau	20	KY400436
	Huila	CoHui	24	KY400468
	Risaralda	CoRis	40	KY400476
Mexico	Chiapas	MxChi	54	KY400491
	Tapachula	MxTap	16	MH806414
	Quintana Roo	MxQro	17	MH806413
	Teocelo	MxTeo	18	MH806417
	Apazapan	MxApa	19	MH806416
	La Jicayana	MxJic	20	MH806415
Guatemala	Guatemala City	GuCit	15	MH806418

(*) In red are sequences from Sutton et al. [32].

**Table 3:** Collection sites, codes, and number of ITS1 sequences by morphotype and country.

		Collection	Sequence	
Morphotype	Country		Code	N
Brazilian-1	Brazil	Uberlândia	B1Ube	10
		Botucatu	B1Bot	10
		Santa Isabel	B1Isa	10
	Argentina	Misiones	ArMis	2
		Concordia	ArCon	3
		Horco-Molle	ArHmo	3
		Tucumán	ArTuc	3
Brazilian-2	Brazil	Santa Isabel	B2Isa	10
		Tres Rios	B2Tri	10
		Ubatuba	B2Uba	10
Brazilian-3	Brazil	Santa Isabel	B3Isa	10
		S.L.Paraitinga	B3Slp	10
		Ubatuba	B3Uba	10
Peruvian	Peru	La Molina	PeLmo	3
		Piura	PePiu	2
	Ecuador	Guayaquil	EcGua	10
Andean	Colombia	Ibagué	CoIba	2
Mexican	Guatemala	Guatemala City	GuCit	2
	Mexico	Tapachula	MxTap	2
		Quintana-Roo	MxQro	3
		Teocelo	MxTeo	4
		Apazapan	MxApa	2
		La Jicayana	MxJic	2

**Table 4:** Classification matrix of individuals by morphotype according to predictive model. Observed values in the rows and the expected in the columns.

	% Correct Classification	Bra-1	Bra-2	Bra-3	And	Mex	Per	Observed
Brazilian-1	98.8	85	0	1	0	0	0	86
Brazilian-2	100.0	0	11	0	0	0	0	11
Brazilian-3	100.0	0	0	21	0	0	0	21
Andean	100.0	0	0	0	15	0	0	15
Mexican	98.4	0	0	0	0	60	1	61
Peruvian	100.0	0	0	0	0	0	45	45
Expected	99.2	85	11	22	15	60	46	239

## Supplementary Materials

The following are available online at https://www.mdpi.com/2075-4450/10/11/408/s1, Figure S1: Fast Minimum Evolutionary inference of the ITS1 sequences of the AF complex; Table S1: Genetic distance among ITS1 sequences from the Brazilian morphotypes; Table S2: Genetic distance among ITS1 sequences from the Mexican morphotype; Table S3: Genetic distance among the ITS1 sequences from the Peruvian morphotype; Table S4: Genetic distance among ITS1 sequences from the Andean morphotype.
