# Supplementary material for: What Can Integrated Analysis of Morphological and Genetic Data Still Reveal about the Anastrepha fraterculus (Diptera: Tephritidae) Cryptic Species Complex?
Source: Insects. 2019 Nov 15;10(11):408. doi: 10.3390/insects10110408 (PMC6921064; doi:10.3390/insects10110408)
Supplement: Supplementary file 1 [file insects-10-00408-s001.pdf]

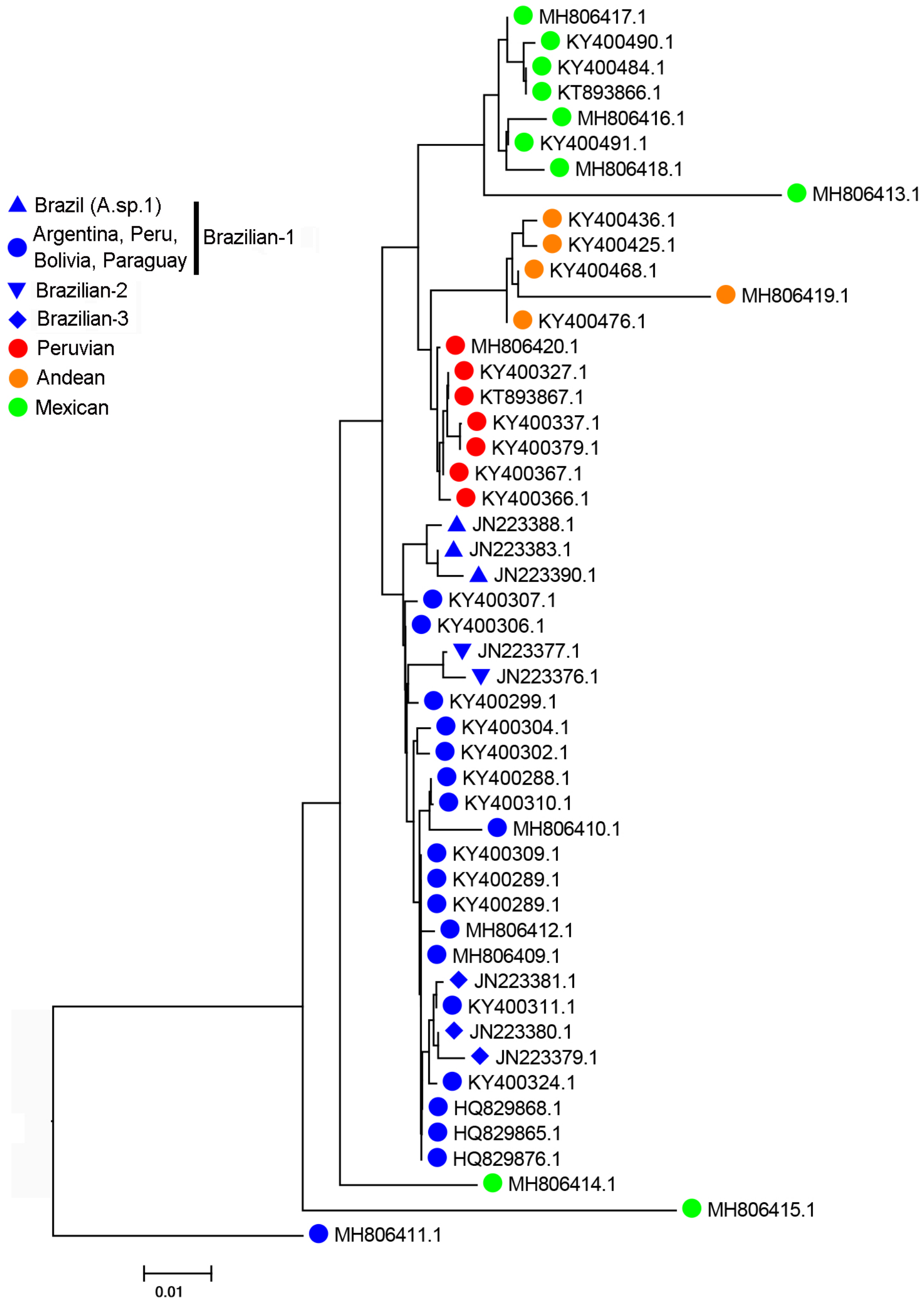

Fig. S1. Phylogenetic analysis by Fast Minimum Evolution method. The evolutionary history was inferred based on genetic distance estimated by Jukes-Cantor method. The tree topology was provided directly by the NCBI site.

Table S1: Genetic distances among ITS1 sequence of the AF complex collected in the highlands of Peru, Bolivia, Argentina and in the inland plateau of eastern Brazil

Br09_PalCha_KY400289																				
Pe66_AyaHua_KY400311	0,000																			
Pe70_CusSil_KY400307	0,002	0,002																		
Pe70_CusSil1_KY400306	0,000	0,000	0,002																	
Pe64_AyaPer_KY400300	0,000	0,000	0,002	0,000																
Pe65_AyaPuc_KY400299	0,000	0,000	0,002	0,000	0,000															
Par_MisSR_HQ829876	0,000	0,000	0,002	0,000	0,000	0,000														
Par_Conc_HQ829868	0,000	0,000	0,002	0,000	0,000	0,000	0,000													
Par_Conc1_HQ829865	0,000	0,000	0,002	0,000	0,000	0,000	0,000	0,000												
Pe68_CusYan_KY400304	0,000	0,000	0,002	0,000	0,000	0,000	0,000	0,000	0,000											
Pe63_AyaHua_KY400324	0,000	0,000	0,002	0,000	0,000	0,000	0,000	0,000	0,000	0,000										
Bol_SC_KT829864	0,002	0,002	0,004	0,002	0,002	0,002	0,002	0,002	0,002	0,002	0,002									
Pe_SENASA_KY400309	0,000	0,000	0,002	0,000	0,000	0,000	0,000	0,000	0,000	0,000	0,000	0,002								
Pe96_VRAE_REg_KY400303	0,000	0,000	0,002	0,000	0,000	0,000	0,000	0,000	0,000	0,000	0,000	0,002	0,000							
Bol_SCVol_KY400288	0,000	0,000	0,002	0,000	0,000	0,000	0,000	0,000	0,000	0,000	0,000	0,002	0,000	0,000						
Pe95_VRAEQan_KY400302	0,000	0,000	0,002	0,000	0,000	0,000	0,000	0,000	0,000	0,000	0,000	0,002	0,000	0,000	0,000					
Pe64_AyaHua_KY400312	0,000	0,000	0,002	0,000	0,000	0,000	0,000	0,000	0,000	0,000	0,000	0,002	0,000	0,000	0,000	0,000				
Pe_SenVRAE2_KT829865	0,000	0,000	0,002	0,000	0,000	0,000	0,000	0,000	0,000	0,000	0,000	0,002	0,000	0,000	0,000	0,000	0,000			
Pe64_AyaPon_KY400298	0,004	0,004	0,007	0,004	0,004	0,004	0,004	0,004	0,004	0,004	0,004	0,007	0,004	0,004	0,004	0,004	0,004	0,004		
Pe91_VRAEReg_KY400310	0,000	0,000	0,002	0,000	0,000	0,000	0,000	0,000	0,000	0,000	0,000	0,002	0,000	0,000	0,000	0,000	0,000	0,000	0,004	
Br1Isa	0,002	0,002	0,004	0,002	0,002	0,002	0,002	0,002	0,002	0,002	0,002	0,004	0,002	0,002	0,002	0,002	0,002	0,002	0,007	0,002
Br1Ube	0,009	0,009	0,011	0,009	0,009	0,009	0,009	0,009	0,009	0,009	0,009	0,011	0,009	0,009	0,009	0,009	0,009	0,009	0,013	0,009
Br1Bot	0,004	0,004	0,007	0,004	0,004	0,004	0,004	0,004	0,004	0,004	0,004	0,007	0,004	0,004	0,004	0,004	0,004	0,004	0,009	0,004
ArCon	0,000	0,000	0,002	0,000	0,000	0,000	0,000	0,000	0,000	0,000	0,000	0,002	0,000	0,000	0,000	0,000	0,000	0,000	0,004	0,000
ArHmo	0,007	0,007	0,009	0,007	0,007	0,007	0,007	0,007	0,007	0,007	0,007	0,009	0,007	0,007	0,007	0,007	0,007	0,007	0,011	0,007
ArMis	0,000	0,000	0,002	0,000	0,000	0,000	0,000	0,000	0,000	0,000	0,000	0,002	0,000	0,000	0,000	0,000	0,000	0,000	0,004	0,000
ArTu5	0,000	0,000	0,002	0,000	0,000	0,000	0,000	0,000	0,000	0,000	0,000	0,002	0,000	0,000	0,000	0,000	0,000	0,000	0,004	0,000
ArTuc	0,133	0,133	0,130	0,133	0,133	0,133	0,133	0,133	0,133	0,133	0,133	0,137	0,133	0,133	0,133	0,133	0,133	0,133	0,138	0,133
Br2Uba	0,002	0,002	0,004	0,002	0,002	0,002	0,002	0,002	0,002	0,002	0,002	0,004	0,002	0,002	0,002	0,002	0,002	0,002	0,007	0,002
Br2Tri	0,004	0,004	0,007	0,004	0,004	0,004	0,004	0,004	0,004	0,004	0,004	0,007	0,004	0,004	0,004	0,004	0,004	0,004	0,009	0,004
Br2Isa	0,004	0,004	0,007	0,004	0,004	0,004	0,004	0,004	0,004	0,004	0,004	0,007	0,004	0,004	0,004	0,004	0,004	0,004	0,009	0,004
Br3Slp	0,000	0,000	0,002	0,000	0,000	0,000	0,000	0,000	0,000	0,000	0,000	0,002	0,000	0,000	0,000	0,000	0,000	0,000	0,004	0,000
Br3Uba	0,000	0,000	0,002	0,000	0,000	0,000	0,000	0,000	0,000	0,000	0,000	0,002	0,000	0,000	0,000	0,000	0,000	0,000	0,004	0,000
Br3Isa	0,000	0,000	0,002	0,000	0,000	0,000	0,000	0,000	0,000	0,000	0,000	0,002	0,000	0,000	0,000	0,000	0,000	0,000	0,004	0,000

0,007												
0,002	0,004											
0,002	0,009	0,004										
0,009	0,016	0,011	0,007									
0,002	0,009	0,004	0,000	0,007								
0,002	0,009	0,004	0,000	0,007	0,000							
0,137	0,142	0,140	0,133	0,145	0,133	0,133						
0,004	0,011	0,007	0,002	0,009	0,002	0,002	0,137					
0,007	0,013	0,009	0,004	0,011	0,004	0,004	0,140	0,002				
0,007	0,013	0,009	0,004	0,011	0,004	0,004	0,140	0,002	0,004			
0,002	0,009	0,004	0,000	0,007	0,000	0,000	0,133	0,002	0,004	0,004		
0,002	0,009	0,004	0,000	0,007	0,000	0,000	0,133	0,002	0,004	0,004	0,000	
0,002	0,009	0,004	0,000	0,007	0,000	0,000	0,133	0,002	0,004	0,004	0,000	0,000

Table S2. Genetic distances among ITS1 sequences from the AF complex collected in Mexico, Guatemala and Colombia

MxChi_KY400491									
MxApa	0,002								
VeCar_KY400490	0,002	0,004							
MxJic	0,150	0,154	0,144						
MxTap	0,044	0,047	0,041	0,126					
MxQro	0,082	0,085	0,086	0,333	0,161				
MxTeo	0,000	0,002	0,002	0,150	0,044	0,082			
GuCit	0,002	0,004	0,004	0,154	0,047	0,085	0,002		
CoSan_KY400484	0,000	0,002	0,002	0,150	0,044	0,082	0,000	0,002	
CoSan_KT8893866	0,000	0,002	0,002	0,150	0,044	0,082	0,000	0,002	0,000

Table S3. Genetic distance among ITS1 sequences from AF complex colleted in lowlands of Ecuador, Peru

PeLam_KY400367											
PeCas_KY400351	0,000										
EcGuy_KY400327	0,000	0,000									
EcGuy_KT893867	0,000	0,000	0,000								
PeCas_KY400389	0,000	0,000	0,000	0,000							
PeJeq_KY400337	0,000	0,000	0,000	0,000	0,000						
PeCas_KY400395	0,002	0,002	0,002	0,002	0,002	0,002					
PeTum_KY400379	0,000	0,000	0,000	0,000	0,000	0,000	0,002				
PeCaj_KY400366	0,002	0,002	0,002	0,002	0,002	0,002	0,004	0,002			
PePiu	0,004	0,004	0,004	0,004	0,004	0,004	0,007	0,004	0,007		
PeLmo	0,007	0,007	0,007	0,007	0,007	0,007	0,009	0,007	0,009	0,011	
EcGua	0,000	0,000	0,000	0,000	0,000	0,000	0,002	0,000	0,002	0,004	0,007

Table S4. Genetic distance among ITS1 sequences from the highlands of Colombia

CoIba						
CoHui_KY400468	0,030					
CoRio_KY400422	0,030	0,000				
CoRiz_KY400476	0,030	0,000	0,000			
CoRio_KY400469	0,030	0,000	0,000	0,000		
CoCau_KY400436	0,030	0,000	0,000	0,000	0,000	
CoRio_KY400427	0,030	0,000	0,000	0,000	0,000	0,000
